# Evaluating the financial case for investing in, or divesting from, tobacco investments

**DOI:** 10.1136/tc-2024-058668

**Published:** 2024-10-30

**Authors:** Timothy Evans, Ayesha Sayed, Corné Van Walbeek

**Affiliations:** 1 University of Cape Town School of Economics, Rondebosch, South Africa; 2 FTI Consulting, Cape Town, South Africa; 3 Department of Finance and Tax, University of Cape Town, Rondebosch, South Africa; 4 Research Unit on the Economics of Excisable Products, School of Economics, University of Cape Town, Rondebosch, South Africa

**Keywords:** Public policy, Economics, Tobacco industry

## Abstract

**Background:**

Tobacco Free Portfolios urges institutions to pledge against investing in, and to withhold financial services from, tobacco companies. Their goal is to create a ‘tobacco-free world’. They argue that without financial and investor support, these companies’ operations will become less sustainable.

**Objective:**

To assess the financial rationale for investing in, or divesting from, tobacco companies.

**Methods:**

Using data sourced from Bloomberg from 2008 to 2023, we evaluate historical sales volumes, real revenue, real gross profit per cigarette, stock performance and price-to-earnings trends for nine leading listed global tobacco companies.

**Results:**

Cigarette sales volumes have steadily declined from 2008 to 2023. Despite efforts to diversify towards novel products, revenues from these products remain small, and cigarettes remain the primary revenue source. Excluding inorganic growth, six of the nine companies experienced real revenue declines from 2008 to 2023. Since 2016, many companies experienced declines in real gross profit per cigarette, indicating that they find it increasingly difficult to offset reduced cigarette sales through net-of-tax price increases. Since 2016, all nine tobacco companies’ stocks have substantially underperformed the market. This stands in contrast to the 2008–2016 period, during which all nine companies’ stocks substantially outperformed the market.

**Conclusions:**

Tobacco companies have experienced deteriorating financial performance since 2016, amidst ever-escalating regulation. It remains unclear whether the growth in novel products will be rapid enough to mitigate the decline in cigarette sales. This uncertainty poses heightened risks for investors, and there is a real possibility of continued poor stock performance.

**WHAT IS ALREADY KNOWN ON THIS TOPIC**

- There is a compelling ethical case to divest from tobacco companies, yet publicly available empirical studies on the financial rationale for divestment are limited. Two such studies, conducted in 2018 and 2019, supported divestment from tobacco stocks based on future valuations and historical share price movements, respectively.

**WHAT THIS STUDY ADDS**

- This paper analyses several financial performance trends of tobacco companies’ using publicly available data. Our analysis is aimed at providing investors and non-specialists a broad overview of the financial performance of leading tobacco companies from 2008 to 2023. We find that, since 2016, these companies have largely experienced declines in cigarette sales, real revenue, real gross profit per cigarette and that their stocks have substantially underperformed the market.

**HOW THIS STUDY MIGHT AFFECT RESEARCH, PRACTICE OR POLICY**

- While the ethical arguments to divest from tobacco stocks are undisputed, we find that a potentially compelling financial argument to divest from these stocks exists as well.

## Introduction

 The primary aim of tobacco-control policy is to reduce the demand for, and supply of, harmful tobacco products.^1^ The tobacco industry, specifically the manufacturers of cigarettes and other tobacco products, is often described as the ‘vector’ of the tobacco epidemic.^2^,^4^ Through its marketing activities, and history of obstructing governments’ attempts to reduce tobacco use, the industry is directly responsible for harms caused by smoking. In most countries, the tobacco industry is dominated by a few large multinationals. However, in some countries, notably China, Indonesia, India and Russia, the industry is dominated by large local companies.

Tobacco Free Portfolios (TFP), founded in 2015, advocates for a ‘world free from tobacco’ and aims to achieve this goal by promoting ‘tobacco-free finance’.^5^ TFP advocates for institutions to sign its ‘Tobacco-Free Finance Pledge’, which commits signatories to abstain from investing in tobacco stocks and withholding financial, investment and insurance services from the tobacco industry.^6^ In the long term, reducing the tobacco industry’s access to investment capital and financial support will make its operations more difficult and expensive.

As of August 2024, 210 entities, including banks, pension plans, asset managers and insurers, have signed TFP’s Tobacco-Free Finance Pledge, collectively representing assets under management of over US$18 trillion.^6^ This would have represented approximately 15.6% of global assets under management in 2022 by fee-charging funds (US$115.1 trillion).^7^

Financial institutions are generally not willing to sacrifice financial returns in favour of ethical considerations.^8^ According to this view, investors seek to maximise returns, not make moral judgments, and a company selling a highly addictive, high-margin product in a large and established industry, with high barriers to entry, may seem a good investment.

Across the world, asset managers, banks and other economic agents continue to include tobacco companies in their investment portfolios to bolster their returns, diversify their holdings and collect dividends.^9^ Historically, tobacco companies have been considered sound investments, as they have paid reliable and high dividends, providing investors with a stream of reliable income outside of share price appreciations.^10 11^

Few publicly available empirical studies assess whether or not a financial case exists for investing in, or divesting from, tobacco companies. Asset management and advisory companies occasionally publish high-level financial opinion pieces, often with a short-term focus and sometimes come to contradictory conclusions.^10 11^

Genus Capital Management (Genus), a Canadian wealth management company and a signatory of the Tobacco-Free Finance Pledge, investigated the merits of holding tobacco stocks for financial performance in 2019.^9^ Genus found that over the period 1998–2018, investment portfolios including tobacco stocks did not outperform analogous portfolios excluding tobacco stocks. Further, they found that portfolios excluding tobacco stocks have generally outperformed the market over the period 2012–2018 and that this trend was expected to persist due to tightening regulation and declining demand for tobacco products.

A study prepared for TFP by Maastricht University in 2018 computed the stock values of major tobacco companies for the start of 2018 using a corporate finance valuation model based on three ‘equally likely’ scenarios: (1) a positive industry outlook with modestly increasing revenues, (2) a neutral industry outlook with limited revenue growth and higher operational costs and (3) a negative industry outlook with significant revenue decreases and reduced profitability. Rather than forecasting stock price movements, the model estimated the company’s intrinsic value and the expected stock price, under different scenarios. The actual stock market prices of the selected companies in mid-2018 suggested that the market expected a scenario between the second and third scenarios, aligning with the valuation generated by the corporate finance model for these scenarios.^12^ In reality, the stock prices of the selected companies have significantly decreased since mid-2018, aligning with scenario 3.

The empirical findings, which focused on historical share price movements and valuations, are somewhat outdated and may not provide comprehensive insights into the current financial considerations for investing in, or divesting from, tobacco stocks. Indeed, stock prices are volatile, and it is well known that past stock-price performance is not indicative of future performance. Of course, researchers do not have perfect foresight, and the question of whether one should invest in an industry is speculative and requires one to make reasonable assumptions.

In this paper, we present relevant long-term trends in stock performance and other financial trends for nine of the world’s largest tobacco companies. Ultimately, we aim to provide guidance to answering the question: is there an economic case for investing in, or divesting from, these tobacco companies?

## Methods and data

This paper evaluates financial trends, including historical sales volumes, revenue, gross profit and price-to-earnings trends, for nine tobacco companies, for their respective financial years (FY) 2008–2023 (ie, n=16). Additionally, we evaluate stock performance trends (inclusive of dividends and capital gains) for these companies, for the calendar years 2008–2023. We obtained this data, derived from financial statements, from Bloomberg, a leading provider of financial news, data and analysis, as well as from integrated annual reports.^13^

The companies selected represent 9 of the top 10 listed tobacco companies, based on market capitalisation in FY2022, for which financial statements are available from FY2008 onwards. Specifically, the list includes Philip Morris International (Philip Morris), Altria Group and Vector Group, listed in the USA; British American Tobacco (BAT) and Imperial Brands, listed in the UK; Gudang Garam and PT Hanjaya Mandala Sampoerna Tbk (Sampoerna), listed in Indonesia; Japan Tobacco, listed in Japan; and the Korea Tobacco & Ginseng Corporation (KT&G), listed in South Korea. India/Mumbai-listed ITC Limited, also 1 of the top 10 listed tobacco companies, is omitted, as it has significant business presence in various industries besides tobacco (in FY2022, cigarettes contributed only 46.5% of its revenue).

To allow comparison between the different companies, the financial performances of all non-US-based companies were converted to US dollars (by Bloomberg) using the average exchange rate for the financial or calendar year in question. Annual financial values were adjusted for inflation and presented in 2022 constant terms, using the US Consumer Price Index corresponding to the relevant financial (or calendar) year-end month. Inflation data were sourced from the United States Bureau of Labor Statistics.^14^ To indicate where we use inflation-adjusted values, we use the term ‘real’ before the appropriate variable.

The compound annual growth rate is commonly used to calculate the annual growth rate over a number of years. It is calculated as $\left[{\left(Y_{final}/Y_{initial}\right)}^{\left(\frac{1}{n}\right)}-1\right]$, where *n* is the number of years. This formula considers only the first and last value of the variable in question and can give distorted results if either of these two values differ substantially from the underlying trend values. To avoid this, for trend analysis, we employ a regression approach, namely: [ln (Y_t_)=α+βt+ε_t_], where ln (Y_t_) is the log of the variable in question and t indicates the various years, t=1, 2,…n. The estimated value of β is the instantaneous growth rate. This rate is converted to an annualised growth rate (g) using the formula: g=100×(exp (β)−1). From this annualised growth rate, the underlying longer-term growth rate is calculated using the formula: h=100×((1+g/100)^n^–1).

## Results

**Figure 1:**
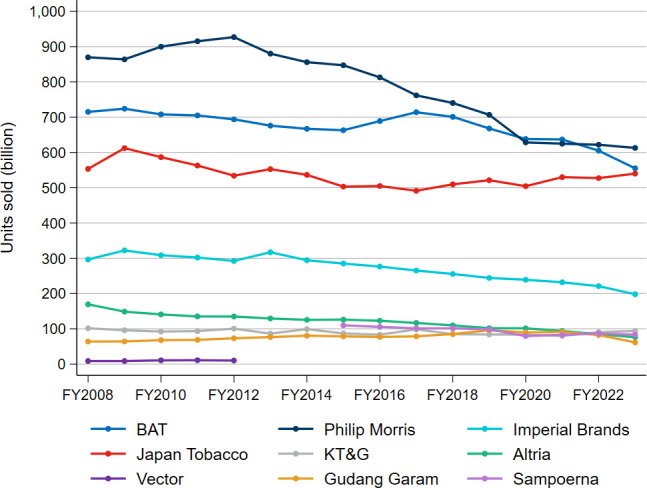
Units sold, FY2008−FY2023. BAT, British American Tobacco; FY, financial year; KT&G, Korea Tobacco & Ginseng Corporation; Philip Morris, Philip Morris International.

Figure 1 illustrates trends in total units sold from FY2008 to FY2023. For BAT, Philip Morris, Altria, KT&G, Gudang Garam and Sampoerna, ‘units sold’ represents the number of cigarettes sticks sold. Imperial Brands reports ‘units sold’ on a stick-equivalent basis to reflect combined cigarette, fine-cut tobacco, cigar and snus volumes. For Japan Tobacco, ‘units sold’ includes both combustible products (eg, cigarettes) and novel products (eg, heated tobacco products (HTPs)), reported on a cigarette-stick equivalent basis. Cigarettes comprised 98.4% of Japan Tobacco’s ‘units sold’ in FY2023. Novel products only accounted for 3.4% of Imperial Brands’ net total tobacco revenue in FY2023. No full data series of units sold exists for either Vector or Sampoerna.

According to the Tobacco Atlas, approximately 4.69 trillion cigarettes were consumed globally in 2023.^15^ Based on data sourced from Bloomberg, as of FY2023, or the latest available data prior to that, the nine companies collectively represent 47.6% (2.23 trillion cigarettes) of this consumption, based on reported ‘units sold’. This percentage has not changed much over the period under consideration, having been 50.1% in FY2008. In FY2023, Philip Morris had a 13.1% share, BAT had 11.8%, Japan Tobacco had 11.5%, Imperial Brands had 4.2%, KTG had 2.0%, Gudang Garam had 1.3%, Sampoerna 1.8% and Vector had 0.2% share. The remaining 2.46 trillion cigarettes were mostly accounted for by the China National Tobacco Corporation (CNTC), a Chinese state-owned monopoly, with annual production estimates ranging between 2.1 and 2.5 trillion.^15^,^17^ Publicly available financial data for CNTC are, however, limited.

Figure 1 indicates a continuous decline in cigarette sales across several of the companies. Using the regression approach from FY2008 to FY2023 Altria’s sales volumes decreased by 49.9%, Philip Morris’s by 37.6%, Imperial Brands’ by 35.9%, BAT’s by 17.2%, Japan Tobacco’s by 11.6% and KT&G’s by 13.1%. In contrast, Gudang Garam’s sales volumes increased by 28.5%. See online supplemental table 1 for details. The sales volumes of the seven companies, for which full data are available, decreased by 25.5% over the 15-year period. However, unlike other companies, Japan Tobacco’s volumes have recently risen by 9.0% from FY2016 to FY2023.

Tobacco companies are increasingly diversifying into novel products such as HTPs and electronic nicotine delivery systems (ENDS). This is, in part, driven by increasing health awareness, tightening regulation and declining cigarette sales. Philip Morris stands out in this transition, and its website notes that its mission is ‘to one day stop selling cigarettes’ as people ‘deserve better alternatives’.^18^ In fact, in 2023 Philip Morris stated its ambition ‘for more than two-thirds of the company’s total net revenues to come from smoke-free products by 2030’.^19^

Of the nine companies, BAT and Philip Morris offer the most detailed historical volume trend data, categorising sales between cigarettes and HTPs. BAT additionally provides volumes for ENDS sales (10 mL units or pods). While cigarettes and HTPs are different products, volumes are comparable as, as with a cigarette, a consumer can draw on an HTP for approximately 6 min or 14 puffs.^20^ ENDS volumes are not comparable to those of cigarettes.

In FY2023, cigarettes constituted 95.9% of BAT’s combined cigarette and HTP unit sales, compared with 99.0% in FY2018. For Philip Morris, the share of cigarettes was 83.0% of combined cigarette and HTP sales in FY2023, down from 94.7% in FY2018. While both companies have been able to substantially increase their HTP sales since FY2018 (using the regression approach, 24.2% per year for Philip Morris and 31.8% per year for BAT), this has not made up for the decline in the sales of cigarettes. In fact, Philip Morris experienced a decline of 43.5 billion units in combined cigarette and HTP sales from FY2018 to FY2023. For BAT, the decline was 129 billion units over the same period.

**Figure 2:**
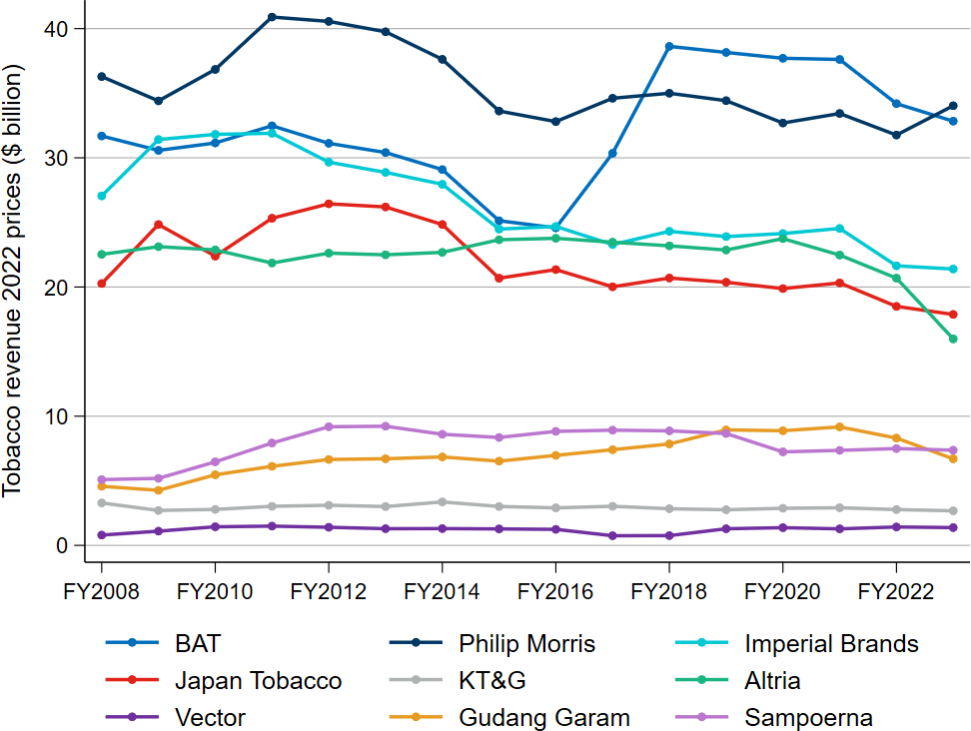
Tobacco companies’ real revenue trends, constant 2022 prices, FY2008–FY2023. BAT, British American Tobacco; FY, financial year; KT&G, Korea Tobacco & Ginseng Corporation; Philip Morris, Philip Morris International.

Figure 2 illustrates the trend in the nine companies’ total real tobacco revenue, including from novel products. Not only have the selected companies failed to generate greater tobacco revenues year-on-year, but many of them also experienced a decline in real revenue. The average annual percentage changes in real revenue are shown in online supplemental table 1.

Using the regression approach, results indicate that Imperial Brands’ real tobacco revenue fell by 32.4% from FY2008 to FY2023, Japan Tobacco’s by 25.6%, Philip Morris’s by 15.5%, KT&G’s by 9.4% and Altria’s by 12.6%. In contrast, Vector’s real revenue increased by 11.3%, Gudang Garam’s by 87.0% and Sampoerna’s by 28.0% (although Sampoerna’s real revenue declined by 23.9% over the period of FY2016–FY2023). BAT’s real revenue increased significantly from FY2016 to FY2018 as a result of its acquisition of Reynolds American (‘Reynolds’) in July 2017, but the overall trend in revenue is negative. For all nine companies combined, again using the regression approach, real revenue decreased by 8.3% from FY2008 to FY2023.

**Figure 3:**
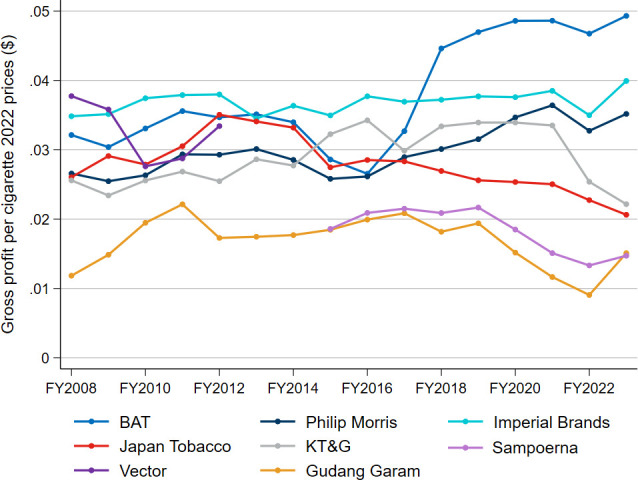
Real gross profit per stick, constant 2022 prices, FY2008–FY2023. BAT, British American Tobacco; FY, financial year; KT&G, Korea Tobacco & Ginseng Corporation; Philip Morris, Philip Morris International.

Real gross profit per cigarette is calculated by dividing total real gross profit by unit sales (figure 3). This measure is imperfect, as some companies report unit sales exclusively for cigarettes, while their gross profits encompass a broader range of products. Nevertheless, since all these companies derive most of their revenue from the manufacture and sale of traditional cigarettes, we believe this is an appropriate proxy for the profitability of cigarettes sold.

Most companies either maintained or increased their real gross profit per cigarette between FY2008 and FY2016 (figure 3). Thereafter, however, several of the companies experienced a decrease in real gross profit per cigarette. Over the period of FY2016–FY2023, with the regression approach, Gudang Garam’s real gross profit per cigarette fell by 51.9%, Japan Tobacco’s by 29.0%, KT&G’s by 30.7% and Sampoerna’s by 43.3%. While BAT and Philip Morris significantly increased their real gross profit per cigarette over this period, this trend may be attributed to increased sales of HTPs and ENDS (reflected in gross profits), rather than their ability to increase profit per cigarette sold amidst volume declines.

Altria’s real gross profit per stick is not shown in figure 3, as it would appear to have almost quadrupled its real gross profit per cigarette during the period to approximately 20 cents, an outcome that seems illogical and likely stems from alterations in reporting of either gross profits or cigarette sales. Ryan and Schafer acknowledge that Altria has historically been able to offset volume declines by increasing cigarette prices, but suggest this model may now be unsustainable with Altria’s operating profit from cigarettes falling in recent quarters.^21 22^

The annual growth rates in real gross profit per stick for the seven companies using the regression approach, for both the 2008–2023 and 2016–2023 periods, are shown in online supplemental tables 1 and 2.

**Figure 4:**
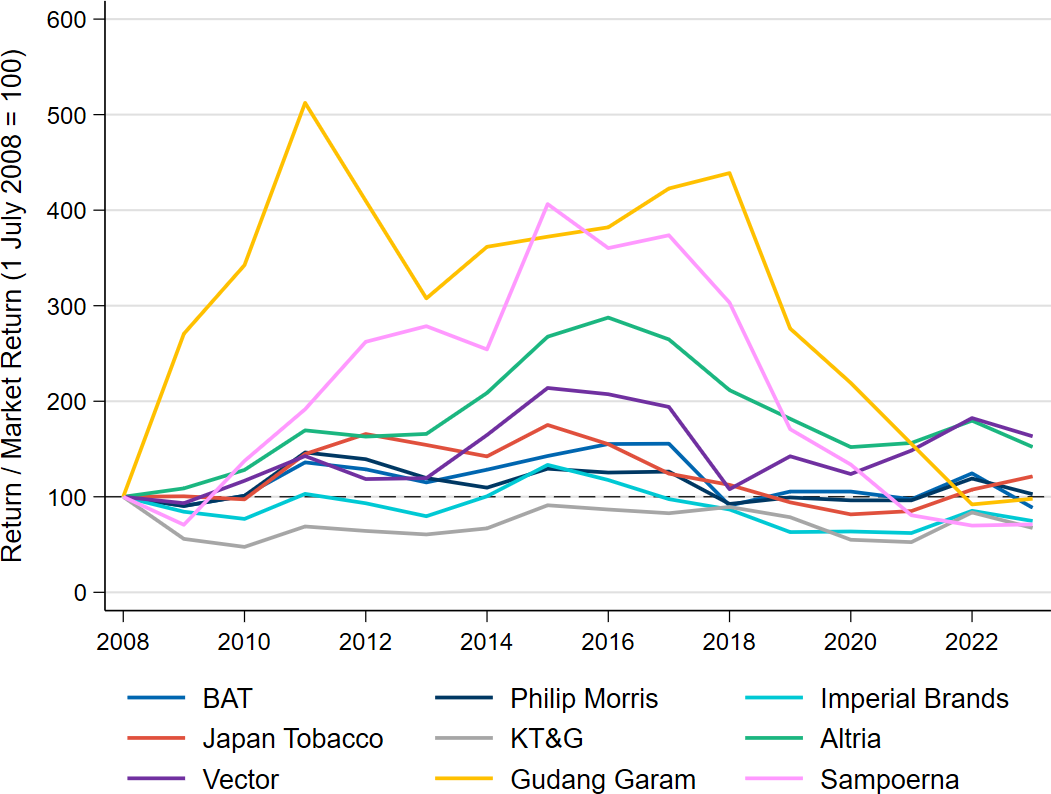
Tobacco stock performance versus market indices performance, 2008–2023. BAT, British American Tobacco; FY, financial year; KT&G, Korea Tobacco & Ginseng Corporation; Philip Morris, Philip Morris International.

Figure 4 illustrates the annual stock performance trends of the selected tobacco companies relative to their respective primary stock exchange indices for the period 2008 to 2023, standardised to 100 in 2008. Stock performance for both the tobacco companies and the stock exchange indices (ie, the denominator) are calculated using price and gross dividend indices from Bloomberg, reflecting both price appreciation and the compounding effect of reinvested dividends within the underlying stock/index. In this context, an increase indicates that the tobacco stock has outperformed its market index, while a decrease indicates underperformance.

BAT’s and Imperial Brands' stock performance is expressed as a proportion of the FTSE All-Share Index, which tracks all stocks traded on the London Stock Exchange. Philip Morris’, Altria’s and Vector’s performance is compared with the New York Stock Exchange Composite Index; Gudang Garam’s and Sampoerna’s to the Jakarta Composite Index; Japan Tobacco’s to the Tokyo Stock Price Index; and KT&G’s to the Korea Composite Stock Price Index.

Over the calendar years 2008 to 2016, tobacco stocks generally outperformed their respective market indices in terms of capital gains and dividends (figure 4). However, in recent years tobacco stocks have significantly underperformed their market indices. For instance, from 2016 and 2023, using the regression approach, the relative performance of BAT declined by 36.9%, Altria by 51.0%, Japan Tobacco by 27.8%, Imperial Brands by 36.9%, KT&G by 29.5%, Gudang Garam by 85.8%, Sampoerna by 89.8%, Philip Morris by 14.1% and Vector by 10.5%. Online supplemental table 3 sets out the actual (dollar) stock performance for these firms over the calendar years 2008 to 2023, adjusted for inflation and standardised to 100 in 2008, for comparison purposes. Online supplemental table 4 does the same, but the figures are standardised to 100 in 2016.

**Figure 5:**
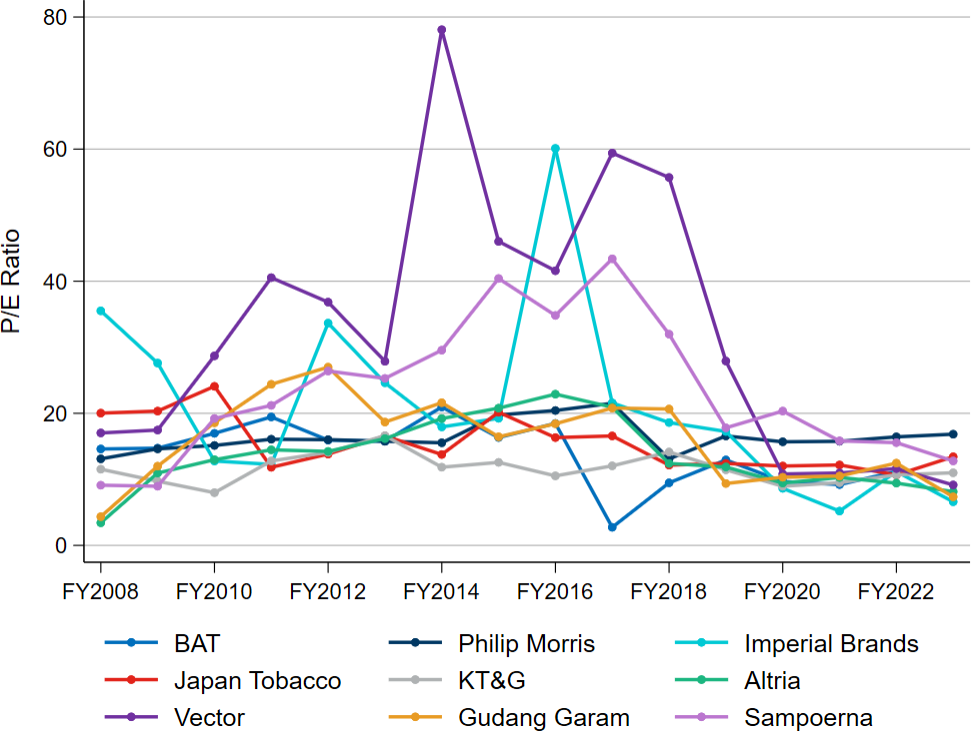
Price/earnings (P/E) ratio trends, FY2008−FY2023. BAT, British American Tobacco; FY, financial year; KT&G, Korea Tobacco & Ginseng Corporation; Philip Morris, Philip Morris International.

Figure 5 depicts the price/earnings (P/E) ratios for the selected companies over the period of FY2008–FY2023. The P/E ratio is a key tool for investors, reflecting the relationship between a company’s stock price and earnings per share, in turn indicating what investors are willing to pay per unit of earnings.

On one hand, the declining P/E ratios of the tobacco companies since 2016 (figure 5) may suggest that the stocks are undervalued (and are therefore a bargain), which would make them good investments for investors. A company trading on a historically low P/E ratio might have potential upside in its share price if it achieves higher-than-expected earnings growth. On the other hand, declining P/E ratios may suggest that business models are in decline, reflecting lower investor confidence in companies’ future prospects and earnings potential.

## Discussion

Since its founding in 2015, TFP has successfully encouraged hundreds of finance companies to divest and withhold from investing in tobacco stocks. Until 2016, the decision to divest from tobacco stocks posed a financial challenge for wealth-maximising investors, given that tobacco stocks typically substantially outperformed the market. However, since 2016, we found that tobacco stocks have generally, on average, performed substantially worse than the market. As a result, portfolios excluding tobacco stocks would probably have outperformed similar portfolios that included tobacco stocks.

Currently, tobacco companies are facing escalating challenges. The WHO reports that, as of 2023, 151 countries (71%) have implemented at least one recommended tobacco-control policy at the best-practice level (eg, health warnings, smoke-free public areas, high taxes, etc). This is a significant increase from 44 countries in 2007.^23^ As a result, the global prevalence of tobacco use (ie, the proportion of the population aged 15+ who use smoked and smokeless tobacco) has declined from 29.3% in 2005 to 21.7% in 2020 and is expected to decrease to 18.1% in 2030.^24^ Similarly, cigarette consumption has declined from 5.9 trillion sticks in 2008 to 4.7 trillion sticks in 2023 (a 20.3% decline).^15^

Our results align with global trends, with cigarette sales for the selected tobacco companies on the decline over the period of FY2008–FY2023, except for Gudang Garam. Collectively, across the selected companies with a full series of units sold data from FY2008 to FY2023, cigarette sales fell from 2.77 trillion sticks to 2.14 trillion sticks (a 22.8% decline).

Our results also indicate that, among the selected tobacco companies, real gross profit per cigarette has peaked and seems to be on the decline, suggesting that these companies are finding it increasingly challenging to offset reduced cigarette sales and escalating excise taxes through net-of-tax price increases.^25^

Moreover, six of the nine selected companies experienced a reduction in real revenues from 2016 to 2023, with Vector, BAT and Gudang Garam being the exceptions. This underscores the difficulty the selected tobacco companies generally face in offsetting declining cigarette sales through the dual strategy of raising net-of-tax cigarette prices and the introduction of novel products. Japan Tobacco, despite increased volumes from 2016 to 2023, also saw reduced revenues over this period, suggesting that higher sales volumes were not enough to offset the decline in the net-of-tax price per stick.

Regarding expansion through novel products, two core challenges confront the selected tobacco companies. First, the growth in the sales of such products is likely to accelerate the decrease in traditional cigarettes sales, further eroding the revenue base of these companies.^26^ This is particularly true, given that novel products still comprise a very small component of the these companies’ revenues. For example, novel products only accounted for 10.3% of BAT’s revenue in FY2023.

Second, the selected tobacco companies face intensified competition in respect of novel products due to the emergence of new multinational competitors. For instance, RLX Technology, a Chinese ENDS firm established in 2018 and listed on the NYSE in 2021, grew its revenues from US$20.1 million in FY2018 to USD 793 million in 2022. Other examples include Smoore International Holdings (founded in 2009), International Vapor Group (founded in 2012) and JUUL (founded in 2015).

Schumpeter’s concept of ‘creative destruction’ appears potentially relevant to the traditional cigarette manufacturing industry, whereby emerging technologies can undermine established companies.^27^ For instance, the stocks of established vehicle manufactures in developed markets such as General Motors, a company which has been slow to innovate, have greatly underperformed that of new entrants in the electric vehicle market like Tesla (although several other factors have driven Telsa’s impressive stock performance). Another example is Nokia, which experienced a significant decline in its mobile phone market share following the launch of the Apple iPhone in 2007.

Importantly, tobacco control measures (increasingly) seek to curb the consumption of both traditional and novel products. HTPs are typically recognised as products similar to cigarettes and are subject to similar strict regulation. While ENDS products currently face less stringent regulation, to date, 121 countries regulate ENDS in some way and 34 of these have banned sales altogether.^23^ Of the countries that regulate ENDS, 42 have banned their use in all public places, 23 ban their advertising and sponsorship and 4 have banned their flavouring.

Whether the selected tobacco companies can maintain, or increase, the growth rates in the sales of novel products will be critical to their financial futures. Philip Morris, more than any other tobacco company, has indicated that novel products are its future vision.

As it stands, our findings indicate a sharp decline in the stock performance of the selected tobacco companies since 2016, all underperforming relative to market indices. This indicates diminished investor confidence, lower perceived value, weakening financial performance and/or negative market sentiment towards the tobacco industry, or at least in respect of tobacco companies established on the back of conventional combustible tobacco products (eg, cigarettes). While it may be true that these companies are currently simply undervalued, an equally plausible explanation is that the market believes that there has been a fundamental decline in these companies’ business models.

Some analysts classify tobacco stocks as ‘defensive’, in that they provide a consistent and reliable dividend yield derived from the sales of a well-established and addictive product, particularly during recessionary periods. The transition to novel products and escalating regulation in the traditional cigarette market have upset this characterisation. The tobacco industry is in flux, introducing heightened uncertainties and risks for investors. If the industry’s transition from traditional cigarettes to novel products is slower than the industry hopes for, coupled with the ongoing decline in cigarette consumption, the industry could then be described as a ‘sunset industry’, that is, an ageing and declining industry.

The tobacco industry has considerable wealth and influence and will continue to do so for years to come. However, when deciding on whether to buy a tobacco company’s shares, financial analysts typically do not look at the absolute size of the company, but rather its future prospects.

Since 2016, leading tobacco companies have generally experienced declines in cigarette sales, real revenue, real gross profit per cigarette and their stocks have substantially underperformed the market. The tobacco industry’s growth trajectory is uncertain, and as a consequence, the financial rationale for including tobacco stocks in a portfolio is not readily apparent, especially as the ongoing decline in the cigarette business is expected to persist.

### Limitations

We provide historical trends of variables that are used to determine company performance, and extrapolate from that. However, past performance is not always indicative of future performance, and any comment about the future performance of tobacco stocks is therefore speculative. For completeness, we also note that the US dollar performance trends for the non-US-based companies are influenced by exchange rate movements.

This analysis is based on financial statements obtained from Bloomberg. While the financial statements have been approved by auditors, tobacco companies, such as other companies, may have an incentive to conceal information which would be harmful to their business. For instance, there may be incomplete company disclosure, which can materially affect the future value of the company, and could bias any analysis.^28 29^

This analysis primarily centres on established tobacco companies built on the foundation of combustible tobacco products. However, we note that different considerations may apply when evaluating the financial case for investing in, or divesting from, newly established tobacco firms focusing solely on novel products.

While this paper does not cover the full multitude of metrics investors use to make decisions, it nevertheless contextualises the financial performance of established tobacco companies, highlights key challenges currently facing these companies and provides investors and potential investors with the background to make more informed decisions regarding their involvement with tobacco companies.

## Conclusion

For decades, investing in tobacco companies appealed to investors because tobacco stocks delivered robust capital gains and consistent dividends. Despite a persistently hostile operating environment, tobacco companies experienced strong financial performance and their stock performance reflected this. However, this trend has shifted. Specifically, since 2016, tobacco shares have substantially underperformed the market, as tobacco companies have been subjected to increased regulation, which has resulted in declining cigarette consumption and stagnant/decreasing real tobacco revenues. Indeed, the most significant threat to their sustainability is the suite of tobacco control measures, which significantly undermines the industry, and which are expected to continue doing so in future.

The outlook for the tobacco industry is uncertain, and the hostile external environment is unlikely to change. Novel products are seen as the future. However, it remains unclear whether the growth in these novel products will be rapid enough to mitigate the decline in traditional tobacco sales, especially as these novel products are increasingly being regulated.

## Supplementary material

10.1136/tc-2024-058668 online supplemental table 1

## Data Availability

Data are available on reasonable request.
