# Supplementary material for: Evaluating the financial case for investing in, or divesting from, tobacco investments
Source: Tob Control. 2024 Oct 30;35(1):e058668. doi: 10.1136/tc-2024-058668 (PMC12911653; doi:10.1136/tc-2024-058668)
Supplement: online supplemental table 1 [file tc-35-1-s001.pdf]

SUPPLEMENTARY MATERIAL

Supplementary Table 1 Regression Trends, FY2008 – FY2023, n = 16

	Units Sold		Real Revenue		Real Gross Profit Per Stick		Price Earnings		*Novel Sales Volumes	
	**Annual (g) [p-value]**	**Long term (h)**	**Annual (g) [p-value]**	**Long term (h)**	**Annual (g) [p-value]**	**Long term (h)**	**Annual (g) [p-value]**	**Long term (h)**	**Annual (g) [p-value]**	**Long term (h)**
**Altria**	-4.2% [0.00]	-49.9%	-0.8% [0.11]	-12.6%			0.4% [0.90]	5.8%		
**BAT**	-1.2% [0.00]	-17.2%	**1.2% [0.10]	**21.1%	3.3% [0.00]	68.6%	-5.0% [0.09]	-55.9%	31.8% [0.00]	423.4%
**Gundan Garam**	1.6% [0.03]	28.5%	4.0% [0.00]	87.0%	-1.5% [0.26]	-21.5%	-1.8% [0.51]	-25.6%		
**Imperial Brands**	-2.7% [0.00]	-35.9%	-2.4% [0.00]	-32.4%	0.4% [0.09]	6.5%	-7.7% [0.02]	-72.2%		
**Japan Tobacco**	-0.8% [0.01]	-11.6%	-1.8% [0.00]	-25.6%	-1.9% [0.01]	-27.0%	-3.4% [0.00]	-42.8%		
**KT&G**	-0.9% [0.03]	-13.1%	-0.6% [0.08]	-9.4%	1.0% [0.20]	18.0%	-0.5% [0.66]	-7.2%		
**Philip Morris**	-2.9% [0.00]	-37.6%	-1.0% [0.00]	-15.5%	1.9% [0.00]	36.1%	0.9% [0.27]	14.7%	24.2% [0.00]	266.3%
**Sampoerna**			1.6% [0.13]	28.0%			1.4% [0.62]	24.1%		
**Vector**			0.7% [0.61]	11.3%			-5.2% [0.15]	-58%		
**Total**	-1.8% [0.00]	-25.5%	-0.5% [0.11]	-8.3%					25.3% [0.00]	286.8%

**Period is FY2018 to FY2023*

** *One* may incorporate a dummy variable (Dummy = 1 for period FY2017 to FY2023) to account for the period following BAT's acquisition of Reynolds. In such case, (g) would be equal to -2.2% [p-value 0.04], and (h) -28.31%. The effect of the Reynolds acquisition is to increase real revenue by 35.4%.

Supplementary Table 2 Regression Trends, FY2016 – FY2023, n = 8

	Units Sold		Real Revenue		Real Gross profit per stick		Price Earnings		*Performance/Market	
	**Annual (g) [p-value]**	**Long term (h)**	**Annual (g) [p-value]**	**Long term (h)**	**Annual (g) [p-value]**	**Long term (h)**	**Annual (g) [p-value]**	**Long term (h)**	**Annual (g) [p-value]**	**Long term (h)**
**Altria**	-6.2% [0.00]	-40.3%	-4.0% [0.03]	-28.1%			-13.3% [0.00]	-68.2%	-8.5% [0.00]	-51.0%
**BAT**	-3.1% [0.00]	-22.5%	3.1% [0.24]	27.3%	7.9% [0.01]	84.3%	3.4% [0.79]	31.0%	-5.6% [0.10]	-36.9%
**Gundan Garam**	-1.4% [0.54]	-10.9%	0.9% [0.66]	7.5%	-8.7% [0.03]	-51.9%	-12.2% [0.02]	-64.7%	-21.7% [0.00]	-85.8%
**Imperial Brands**	-4.2% [0.00]	-28.8%	-1.6% [0.06]	-11.9%	0.3% [0.68]	2.2%	-24.2% [0.00]	-89.1%	-5.6% [0.11]	-36.9%
**Japan Tobacco**	1.1% [0.00]	9.0%	-2.0% [0.00]	-15.1%	-4.2% [0.00]	-29.0%	-4.2% [0.06]	-29.0%	-4.0% [0.24]	-27.8%
**KT&G**	0.3% [0.84]	2.1%	-1.1% [0.07]	-8.1%	-4.5% [0.06]	-30.7%	-2.0% [0.38]	-15.2%	-4.3% [0.19]	-29.5%
**Philip Morris**	-4.2% [0.00]	-29.1%	-0.4% [0.45]	-3.4%	4.1% [0.00]	37.7%	-2.6% [0.31]	-18.9%	-1.9% [0.38]	-14.1%
**Sampoerna**			-3.4% [0.01]	-23.9%	-6.9% [0.00]	-43.4%	-15.5% [0.00]	-73.9%	-24.8% [0.00]	-89.8%
**Vector**			6.9% [0.10]	70.3%			-25.4% [0.00]	-90.4%	-1.4% [0.72]	-10.5%
**Total**	-2.6% [0.00]	-18.9%	-0.7% [0.44]	-5.4%						

** Relative Stock performance is measured based on calendar years, not financial years.*

Supplementary Table 3 Real Stock performance trends, indexed (2008 = 100)

Year	Altria	BAT	Gundam Garam	Imperial Brands	Japan Tobacco	KT&G	Philip Morris	Sampoerna	Vector
**2008**	100.0	100.0	100.0	100.0	100.0	100.0	100.0	100.0	100.0
**2009**	136.6	127.1	588.1	118.5	103.0	92.0	113.4	153.6	117.2
**2010**	180.2	154.7	1,150.9	117.6	112.2	98.1	142.2	462.2	164.3
**2011**	223.5	194.2	1,731.5	147.1	142.7	121.9	193.0	647.7	188.0
**2012**	245.5	212.5	1,491.1	153.9	173.6	133.9	209.8	954.4	178.5
**2013**	310.9	230.5	878.9	159.6	201.4	128.4	224.3	795.7	224.2
**2014**	415.1	242.9	1,260.5	190.2	178.8	130.8	217.9	886.2	327.5
**2015**	507.3	255.9	1,032.1	239.3	242.6	171.2	245.3	1,126.8	405.6
**2016**	598.6	266.9	1,249.0	202.1	218.2	163.8	261.0	1,177.9	431.7
**2017**	641.7	324.6	1,651.4	203.5	216.8	213.9	306.4	1,460.0	470.4
**2018**	459.1	158.6	1,569.8	151.4	165.0	184.1	200.7	1,084.7	234.2
**2019**	484.5	223.1	1,049.3	133.4	161.8	167.8	264.8	649.0	379.8
**2020**	428.4	204.6	793.8	123.5	156.6	164.8	271.4	484.4	349.0
**2021**	497.4	206.9	582.5	131.8	154.3	141.9	306.3	302.3	472.0
**2022**	487.6	222.6	318.6	152.6	157.0	153.6	323.3	242.0	494.9
**2023**	455.0	174.0	365.8	146.8	204.6	140.4	307.2	265.8	488.3

Source: Financial data obtained from Bloomberg

Supplementary Table 4 Real Stock performance trends, indexed (2016 = 100)

Year	Altria	BAT	Gundam Garam	Imperial Brands	Japan Tobacco	KT&G	Philip Morris	Sampoerna	Vector
**2016**	100.0	100.0	100.0	100.0	100.0	100.0	100.0	100.0	100.0
**2017**	107.2	121.6	132.2	100.7	99.4	130.6	117.4	124.0	109.0
**2018**	76.7	59.4	125.7	74.9	75.6	112.4	76.9	92.1	54.2
**2019**	80.9	83.6	84.0	66.0	74.1	102.4	101.5	55.1	88.0
**2020**	71.6	76.6	63.6	61.1	71.8	100.6	104.0	41.1	80.8
**2021**	83.1	77.5	46.6	65.2	70.7	86.6	117.4	25.7	109.3
**2022**	81.5	83.4	25.5	75.5	71.9	93.8	123.9	20.5	114.7
**2023**	76.0	65.2	29.3	72.6	93.8	85.7	117.7	22.6	113.1

Source: Financial data obtained from Bloomberg
